# Jagged1 Is Altered in Alzheimer's Disease and Regulates Spatial Memory Processing

**DOI:** 10.3389/fncel.2017.00220

**Published:** 2017-08-09

**Authors:** Swananda Marathe, Muriel Jaquet, Jean-Marie Annoni, Lavinia Alberi

**Affiliations:** ^1^Department of Medicine, University of Fribourg Fribourg, Switzerland; ^2^Swiss Integrative Center for Human Health SA Fribourg, Switzerland; ^3^Neurology Clinic, Cantonal Hospital Fribourg, Switzerland

**Keywords:** Notch signaling, Jagged1, hippocampus, Alzheimer's disease, memory

## Abstract

Notch signaling plays an instrumental role in hippocampus-dependent memory formation and recent evidence indicates a displacement of Notch1 and a reduction its activity in hippocampal and cortical neurons from Alzheimer's disease (AD) patients. As Notch activation depends on ligand availability, we investigated whether Jagged1 expression was altered in brain specimen of AD patients. We found that Jagged1 expression was reduced in the CA fields and that there was a gradual reduction of Jagged1 in the cerebrospinal fluid (CSF) with the progression of dementia. Given the role of Notch signaling in memory encoding, we investigated whether targeted loss of Jagged1 in neurons may be responsible for the memory loss seen in AD patients. Using a transgenic mouse model, we show that the targeted loss of Jagged1 expression during adulthood is sufficient to cause spatial memory loss and a reduction in exploration-dependent Notch activation. We also show that Jagged1 is selectively enriched at the presynaptic terminals in mice. Overall, the present data emphasizes the role of the Notch ligand, Jagged1, in memory formation and the potential deficit of the signaling ligand in AD patients.

## Introduction

The Notch pathway is one of the most conserved signaling cascades regulating cellular communication, cell fate specification and morphogenesis from development onward (Artavanis-Tsakonas et al., 1999; Huppert et al., 2000; Pierfelice et al., 2011; Guruharsha et al., 2012). In the past two decades, a wealth of studies have demonstrated that Notch signaling is fundamental for neurophysiological processes such as learning and memory in a variety of species, including mammals (Alberi et al., 2013). Our group has previously shown that the loss of Notch1 from the CA1 subfield of the hippocampus of mice results in a significant impairment in LTP and LTD and a considerable deficit in hippocampus-dependent spatial memory. Furthermore, at a mechanistic level, we have shown that in the absence of Notch1, NMDA currents are significantly reduced and downstream activation of cAMP response element binding (CREB) protein is profoundly affected (Brai et al., 2015), likely accounting for the plasticity deficit. The Notch1-CREB axis has been prevously reported in fruit flies, where Notch1 protein has been shown to be essential for CREB hyperphosphorylation and its subsequent activation (Zhang et al., 2013). These works clearly establish Notch signaling as an important modulator in the process of memory encoding (Marathe and Alberi, 2015).

Given that the memory loss is a prominent behavioral symptom of AD, we recently showed that Notch signaling is compromised in the brains of AD patients (Brai et al., 2016), inferring a causal relationship between alterations in Notch1 function and memory impairment, which we further explore in this manuscript.

Notch receptors are activated through ligand binding, which triggers a sequential cleavage by metalloproteases and the γ-secretase complex (Kopan and Ilagan, 2009). The Notch receptors are activated by the Jagged and Delta Serrate family of ligands containing a conserved DSL (Delta/Serrate/Lag-2) domain that is necessary for binding with the receptor (D'Souza et al., 2008). Canonical and non-canonical ligands, like Jagged1 and DNER, have been extensively characterized in the context of Notch signaling and appear to be exclusively involved in activation of this pathway (Alberi et al., 2013). However, since Notch is highly interlinked with other signaling cascades, e.g., Wnt (Hayward et al., 2005) and Reelin (Hashimoto-Torii et al., 2008), Notch ligands may have additional roles on cell functions by indirectly influencing other cellular pathways. Our previous study showed that Jagged1 is highly expressed in cultured neurons, whereas the expression of Delta like-1 is negligible. Moreover, Jagged1 colocalizes with presynaptic markers and its expression is increased upon synaptic activation (Alberi et al., 2011). The involvement of Jagged1 in mediating Notch-dependent plasticity was suggested by another study using mice carrying a constitutive heterozygous null allele of Jagged1 (Jagged1^+/−^ mice), which displayed a significant deficit in hippocampus-dependent spatial memory (Sargin et al., 2013) resembling Notch1 depletion (Costa et al., 2003; Alberi et al., 2011). Although, a potential neurodevelopmental deficit in the Jagged1^+/−^ mice can not be excluded as contributing factor to the behavioral deficit, the existing data suggest that Jagged1 might be the most relevant Notch ligand to be investigated in the context of memory impairment and AD pathophysiology. In this paper, we show that Jagged1 expression is significantly and progressively affected in AD, we demonstrate that conditional loss of Jagged1 in hippocampal neurons affects memory formation and activity-dependent Notch signaling. Lastly, we confirm that Jagged1 is enriched presynaptically in hippocampal neurons from the mouse brain. We believe that investigation of Notch ligands involved in memory formation is critical in order to better understand the role of Notch signaling in AD and ultimately devise more effective therapeutic strategies against dementia.

## Material and methods

### Human tissue

The human samples were generously provided from the Brain Bank for Dementia Research, Oxford, UK. We received paraffin embedded brain sections from the entorhinal cortex of 5 controls and 5 sporadic AD patients and frozen CSF from a second group of 6 non-demented controls and 6 AD patients (Table 1). CSF from living patients was obtained through the Memory Clinic of the Cantonal Hospital of Fribourg (Table 1). The use of human postmortem tissue has been approved by the Ethical commission of the Brain Bank for Dementia UK [OBB ID N. TW344, OBB ID N. TW296 and the Swiss Ethical Commission (CER-VD N. 2014-325)]. The use of *ex-vivo* human specimen for basic research purposes is licensed by Swiss Ethical Commission (CER-VD N.2016-01627). All experiments conducted on human tissue comply with the WMA Declaration of Helsinki.

**Table 1:** Human specimen.

**Patient ID**	**Age**	**Sex**	**Braak staging (Braak and Braak, 1991)**	**CERAD**	**Tissue**	**Applications**
035/13	85	m	5	Definite AD	EC/HP	IHC
016/12	64	f	6	Definite AD	EC/HP	IHC
089/11	83	f	6	Definite AD	EC/HP	IHC
133/12	78	m	6	Definite AD	EC/HP	IHC
100/12	90	m	4	Probable AD	EC/HP	IHC
1117/00	91	f	6	Definite AD	CSF	WB
C4295	84	m	6	Definite AD	CSF	WB
142/05	87	m	6	Definite AD	CSF	WB
C4298	76	m	5-6	Definite AD	CSF	WB
C4130	79	f	5-6	Definite AD	CSF	WB
036/02	69	f	6	Definite AD	CSF	WB
169/11	84	f	2	Normal	EC/HP	IHC
071/13	83	f	2	Normal	EC/HP	IHC
136/12	77	f	1	Normal	EC/HP	IHC
121/12	89	f	2	Normal	EC/HP	IHC
048/12	65	f	1	Normal	EC/HP	IHC
048/02	89	m	1	Normal	CSF	WB
1085/03	67	m	1	Normal	CSF	WB
053/06	71	m	1	Normal	CSF	WB
083/01	79	f	0	Normal	CSF	WB
1050/00	81	f	1	Normal	CSF	WB
1182/94	80	f	1	Normal	CSF	WB
**Patient ID**	**Age**	**Sex**	**MMSE**	**CDR**	**Tissue**	**Applications**
145/14	73	m	26/30	0	CSF	WB
530/14	74	f	29/30	0	CSF	WB
907/14	79	f	20/30	0	CSF	WB
569/15	65	m	30/30	0	CSF	WB
860/15	65	m	28/30	0	CSF	WB
462/15	64	m	15/30	1.5	CSF	WB
204/14	76	f	22/30	1	CSF	WB
845/15	79	f	22/30	1	CSF	WB
777/15	80	m	21/30	1.5	CSF	WB
155/15	84	m	19/30	1.5	CSF	WB

*CERAD, Consortium to Establish a Registry for Alzheimer's Disease; HP, Hippocampus; EC, Entorhinal Cortex; MMSE, Minimal Mental State Evaluation; CDR, Clinical Dementia Rating*.

### Mice

Mouse behavior experiments were performed with permission of the local animal care committee (Canton of Fribourg, Switzerland) and according to the present Swiss law and the European Communities Council Directive of November 24 1986 (86/609/EEC). Mice were housed with littermates in standard IVC mouse cages in a temperature and humidity controlled animal room. All mice were maintained on a 12-h light/dark cycle and all testing was performed during the light phase. Food and water were available *ad libitum*.

Jagged1^flox/flox^ mice (Nyfeler et al., 2005) (gift from Dr. Verdon Taylor, UNIBAS) were crossed with the B6;129S6-Tg(Camk2a-cre/^ERT2^)1Aibs/J (Madisen et al., 2010) (Jackson Laboratory) to generate excitatory neuron specific, tamoxifen- inducible Jagged1 conditional knockout (Jagged1cKO) mice. Two months old Jagged1cKO mice were injected i.p. with 67 mg/kg of tamoxifen (TAM) every day for 10 consecutive days. The mice were then left for at least 10 additional days for the existing Jagged1 protein to dissipate.

No Jagged1cKO mouse was treated with TAM prior to 2 months of age to avoid developmental abnormality. Control (CTL) mice were either TAM-injected Tg(Camk2a-cre/^ERT2^) or uninjected Jagged1cKO.

### Immunolabeling

#### Reagents for immunolabeling

Following primary antibodies were used for fluorescent immunohistochemistry on slices: goat anti Notch1 (Santa Cruz Biotechnology, cat. no. SC6014), goat anti Jagged1 (Santa Cruz Biotechnology, cat. no. SC6011), rabbit anti NeuN (Abcam, cat. no. ab177487), mouse anti Hes5 (Abcam, cat. no. ab25374). Misfolded β-sheets were stained with Thioflavin-T (Abcam, cat.n. ab120751) at a concentration of 100 mM diluted in water, following the manufacturer's instructions. Nuclei were counterstained with DAPI (Thermo Fisher Scientific). The primary antibodies used for the chromogen immunohistochemistry on mouse brain sections were rabbit anti Jagged1 (Abcam, cat. no.ab7771) and goat anti-Notch1(sc-6014; Santa Cruz Biotechnology, USA). The rabbit anti Jagged1 (Abcam, cat. no.ab7771) was used for immuno-electronmicroscopy (ImmunoEM). Antibodies used for Western blot analysis were: rabbit anti Jagged1 (Abcam, cat. no. ab7771), mouse anti Jagged1 (Novus Biologicals, cat. no. H00000182-M01A), rabbit anti DNER (Abcam, cat. no. ab174484), and mouse anti GAPDH (Santa Cruz Biotechnology cat. no. SC365062). The secondary antibodies used for the immunofluorescence were Cy2, Cy3, or Cy5 conjugated donkey anti-goat, Cy2 and Cy3 conjugated donkey anti-rabbit, Cy3 donkey anti-mouse. The secondary antibodies for the chromagen immunohistochemistry were biotinylated donkey anti rabbit and biotinylated donkey anti rabbit. All fluorescent or biotynilated conjugated antibodies were purchased from Jackson Immunoresearch Europe Ltd and were diluted 1:500 for fluorescent immunohistochemistry and 1:2,000 for fluorescent immunoblots.

#### Immunohistochemistry

Paraffin sections from humans and floating mouse sections were processed either for fluorescent or chromagen immuno-histochemistry according to the previously published protocols (Brai et al., 2015; Marathe et al., 2015). For fluorescent immunolabelings, DAPI was used for nuclear counter-staining. Fluorescent immunolabeled section where imaged at the confocal microscope (Zeiss LSM 700). Chromagen immmunolabeled sections were imaged using an optical slide scanner (Nanozoomer Hamamatsu 2.0 HT). The fluorescence intensity (integrated pixel density) of the immunolabeled proteins on human and mouse brain sections were measured on Region of Interest (ROI) drawn on the perimeter of the cell bodies, using the ROI manager and particle analysis tool of ImageJ (https://imagej.nih.gov/ij/).

#### Immuno electron microscopy and quantitation

The mice were transcardially perfused with 4% PFA and 0.1% glutaraldehyde. The Immunoelectromicroscopy (IEM) was carried out on hippocampal sections from 3 WT mice using the Rabbit anti Jagged1 primary antibody (Abcam, cat. no.ab7771) according to the published protocol (Brai et al., 2016). For particles quantification, the size of each 8-bit image was calibrated according to the magnification indicated by the scale bar. The particle were separated from the background by adjusting the threshold for pixel identification within the grayscale range of 0–70. For delimiting the pixel counts on presynaptic and postsynaptic areas, ROIs were drawn manually following the visible presynaptic and postsynaptic membranes and closed at the top or bottom to form confined areas, which were measured using the Measure function of ImageJ (presynaptic area: 82.3 ^*^ 10^3^ ± 66.1 ^*^ 10^3^ nm^2^, postsynaptic area: 57.3 ^*^ 10^3^ ± 32.8 ^*^ 10^3^ nm^2^; *t*_40_ = 0.89, *p* = 0.38, *n* = 21 presynaptic and *n* = 21 postsynaptic areas). Particles were counted automatically by setting the size within the range: 1.40-Infinity and circularity within the range: 0.10–1.00. The number of resulting particles was divided by the value of the presynaptic or postsynaptic area in nm^2^.

#### Immunoblotting

Western blot on tissue lysates and CSF was performed as previously described (Marathe et al., 2015; Brai et al., 2016). Visualization was performed through a fluorescent imager (Omega Lum G from Aplegen). The optical density of each band was assessed through ImageJ, using the gel tool application and bands were normalized to GAPDH.

### Behavioral tests

Jagged1cKO and CTL mice were used in all behavioral tests. To control for the potential off-target effect of TAM on cognitive performance, the majority of CTL mice were TAM-injected Tg(Camk2a-cre/^ERT2^). To assess whether TAM may influence behavior, Anxiety behavior was examined between CTL groups, TAM-injected Tg(Camk2a-cre/^ERT2^) and uninjected Jagged1cKOs, using the open field test [TAM-injected Tg(Camk2a-cre/^ERT2^): 309.65 ± 6.15 s, non injected Jagged1cKO : 325.10 ± 29.20 s; *F*_(1, 7)_ = 0.23, *p* = 0.62, *n* = 4 animals per genotype]. Differences in hippocampus-dependent memory were tested using Novel Object Displacement (NOD) test [TAM-injected Tg(Camk2a-cre/^ERT2^): 87.87 ± 7.98%, non injected Jagged1cKO : 81.67 ± 15.66%; *F*_(1, 10)_ = 0.22, *p* = 0.8, *n* = 6 animals per genotype].

Mice were habituated to the behavior room for at least three days before the beginning of the behavioral tests and were handled daily by the experimenter during this period.

To test the unprovoked locomotor behavior and baseline anxiety levels, mice were introduced in an open arena (60 X 60 cm square box) and were allowed to explore freely for 10 min. The video was recorded using a webcam mounted on top of the arena and the movement of mice was tracked using an ImageJ plugin OFT (https://cbsn.neuroinf.jp/modules/xoonips/detail.php?id=ImageOF). The average speed was calculated during the period when mice were actively moving, while the periods where they were immobile were discarded. Also, time spent and the distance traveled in the central quadrant was quantified.

The conditional knockout mice and the CTL mice were also tested on an elevated plus maze, in which mice were allowed to explore a plus-shaped maze with two open and two closed arms. The maze was elevated 3 feet from the ground, so that the open (un-walled) elevated arms create an anxiogenic atmosphere. The video was recorded using a webcam mounted on top of the arena and the movement of mice was tracked using an ImageJ plugin EPM (https://cbsn.neuroinf.jp/modules/xoonips/detail.php?id=ImageEP). Time spent in the open arms as a percentage of total time was quantified to assess anxiety behavior.

Mice were tested for hippocampus-dependent memory behavior on the Y-maze (hidden arm version) test and the NOD test. Y-maze was performed as described previously (Alberi et al., 2011) in a Y-shaped maze with three walled arms (height: 12.7 length: 38.3, width: 7.6 cm) at 120^0^ angle from one another. The animals were placed at the center of the maze, facing the wall and were left to explore the Y maze for 5 min. Spontaneous alternation percentage was scored. The next day, one arm was blocked (hidden arm, C) and the animals were allowed to explore the remaining arms for 5 min. Twenty minutes after the 2-arm exploration, all arms were left open and mice were reintroduced in the Y maze for another 5 min. The mice were videotaped and scored for number of entries and time spent in each arm using stopwatch+ software (http://www.cbn-atl.org/research/stopwatch.shtml).

The NOD test was performed in a rectangular arena, to which the mice were first habituated for 3 consecutive days for 15 min each. On 4th day, four objects of same material but of distinctly different shapes and texture were placed at four corners of the arena and the animals were allowed to explore the arena with the four objects for 5 min. On 5th day, the animals again encountered the same four objects for 5 min, except that two of them had swapped positions. The behavior was recorded using a video camera mounted on top of the arena and the behavior was analyzed *post-hoc* from the videos. The object-location discrimination index was calculated as (Time spent with displaced objects / Total Time spent exploring all objects)^*^100. The object-location discrimination index of 50% indicated lack of discrimination.

For studying protein expression changes following novel environmental exploration, mice were taken from their home cage and introduced into a rectangular arena containing 2 objects of distinct color and texture. Mice were allowed to explore the arena for 10 min after which they were returned to their home cage. Forty-five minutes after exploration, mice were sacrificed by transcardial perfusion with 4% PFA to study protein expression by immunohistochemistry as shown previously (Alberi et al., 2011; Brai et al., 2015).

### Statistical analysis

Statistical comparison of Jagged1 expression in hippocampal neurons of post-mortem sections from healthy controls and AD patients was obtained using one-way Anova with *post-hoc* Bonferroni correction. Optical density analysis of bands on Western Blot from human and mouse samples were analyzed by using paired Student's *t*-test to assess a significant difference between two groups. The fluorescence intensity of Notch1 and Hes5 was analyzed from randomly picked hippocampal neurons and results between genotype compared by non-parametric Mann Whitney Test. Behavioral differences between KOs and CTL mice were quantified using one-way Anova with *post-hoc* Bonferroni correction. For IEM analysis, comparison between Jagged1 immunogold particles on the area of the the pre- and post-synaptic terminal was carried out using one-way Anova with *post-hoc* Bonferroni correction. All statistical analysis were conducted using the Real Statistics add-in in Excel from Charles Zaiontz (http://www.real-statistics.com/). The differences were considered to be significant when the *p*-value was less than 0.05.

## Results

### Jagged expression in hippocampi from AD patients

**Figure 1:**
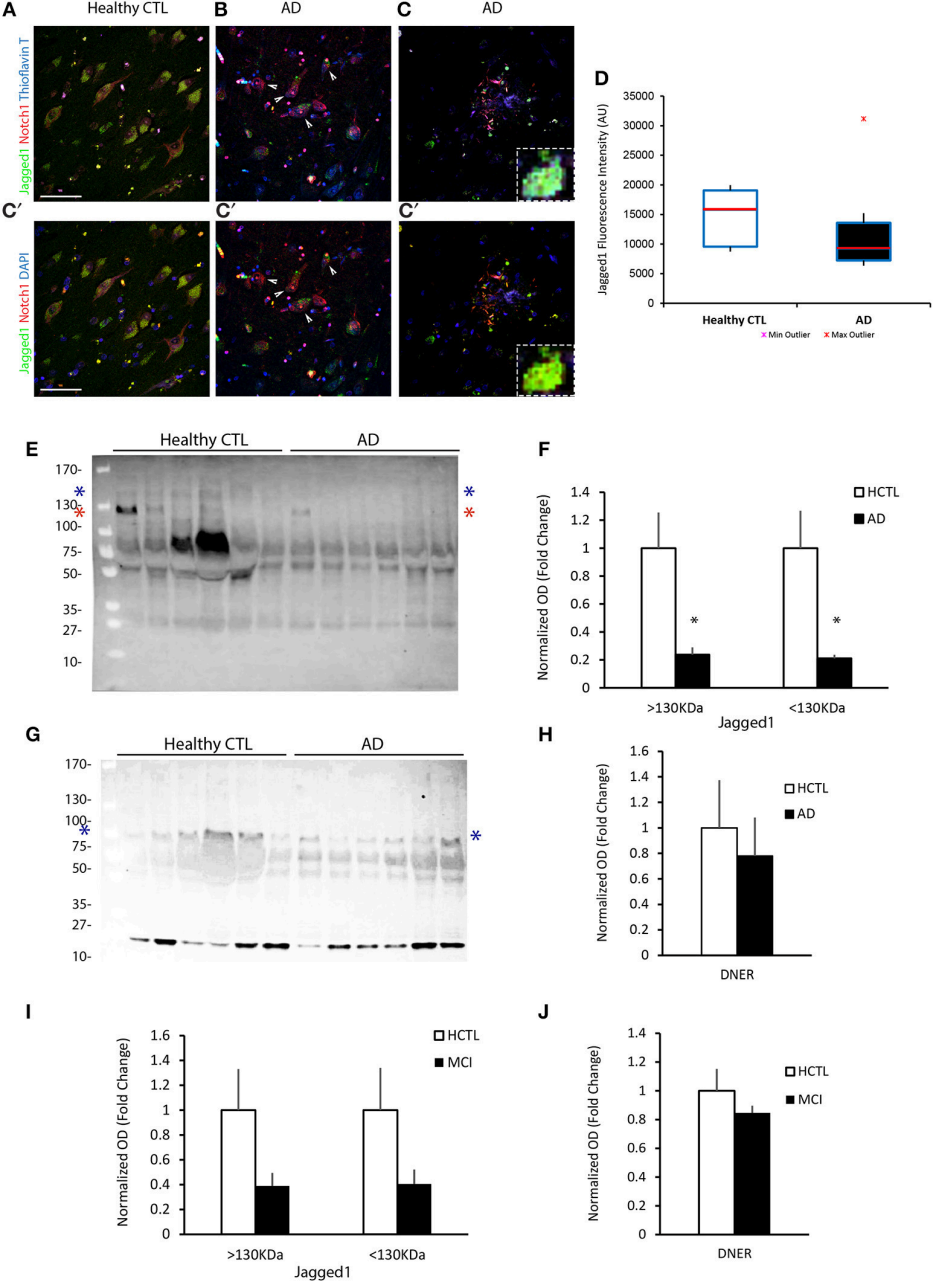
Jagged1 expression in brains and CSF of AD patients. Representative double fluorescent immunolabelings for Jagged1 (green) and Notch1 (red) counterstained with Thioflavin-T or DAPI (blue) on postmortem brain sections comprising the hippocampal CA fields from healthy age-matched controls and AD patients **(A–C)**. In the healthy controls, Jagged1 is localized to the somata of neurons where also Notch1 is expressed. As expected, Thioflavin-T labeling is negligible **(A,A′)**. AD sections show fibrillary aggregates **(B,B′)** and **(C,C′)** core plaques double positive for Notch1 and Thioflavin-T. Jag1 expression is scattered in the parenchyma and low in, degenerated neurons (white arrows) **(B,B′)**. Jag1 overlays in small double positive aggregates for Notch1 and Thioflavin-T (100x magnification) in radiating plaques with a visible reduction in Jagged1 cellular expression **(B–C′)**. Box plots summarizing the quantification of the fluorescence intensities of Jagged1 immunolabeled neurons shows a significant reduction in Jagged1 expression in AD patients (*p* < 0.001) **(D)**. Immunoblotting on CSF from 6 patients per condition reveals that Jagged1 bands over 130 KDa corresponding to the full length (blue star) and the one below 130 KDa (red star) indicating the soluble Jagged1 appear less represented in the CSF from AD patients **(E)**. Bar graph showing the pattern of expression of Jagged1 bands >130 KDa (blue stars) and <130 KDa (red stars) **(F)**. Representative immunoblotting of the CSF from the same patients with DNER shows the expected band <100 KDa (blue stars) and a stronger undefinded band <27 KDa. Intenstity of the bands at both molecular weights show a high variability independently of the condition **(G)**. Bar graph summarizes the expression of the expected DNER band <100 KDa in CTLs and ADs **(H)**. Bar graphs representing the quantification of the Jag1 bands >130 and <130 KDa and the DNER band <100 KDa in MCI patients and age-matched CTLs **(I,J)**. Scale bars are 25 μm in **(A,A′)**. All graphs represent mean ± SEM, ^*^p < 0.05. AU, arbitrary units.

We have previously shown that Notch1 is significantly altered in the hippocampus and entorhinal cortex of AD patients, with reduced expression in pyramidal neurons as well as decreased Notch1 activity (Brai et al., 2016). Based on the well-established role of Notch1 in memory formation across species, we investigated whether the cognate ligand Jagged1, which is expressed in neurons and can be induced by synaptic activity (Alberi et al., 2011), was also altered in post-mortem specimens from patients with severe dementia (Table 1) and *ex-vivo* CSF samples from patients with mild cognitive impairment (MCI) based on the minimal mental score evaluation (MMSE; Table 1). Our immunohistochemical analysis on post-mortem sections comprising the hippocampus and entorhinal cortex (Figure S1A), reveals that in AD patients the expression of Jagged1 is substantially altered as compared to age-matched healthy controls (Healthy CTLs; Figures 1A–C′). Moreover, Jagged1 expression is evident in pixels in the parenchyma in and around Thioflavin-T and Notch1 positive aggregates (Figure 1B) and degenerated neurons, as indicated by the condensed nuclei (white arrows, Figures 1B,B′). Jagged1 is present in scattered depositions, around rosette-like plaques, Thioflavin-T and Notch1 positive (Figures 1C,C′; magnified insert). Analysis of Jagged1 immunofluorescence intensity in CA field neurons, defined by the larger appearance of their neurons and their sterotypical orientation, reveals a significant reduction of Jagged1 [Healthy CTLs: 3293.62 ± 196.31, AD: 1969.28 ± 172.50; *F*_(1, 279)_ = 25.69, *p* < 0.001; *n* = 122 neurons analyzed in CTL and *n* = 158 neurons in AD sections, *n* = 5 patients per condition; Figure 1D]. The expression of Jagged1 inversely correlates with the staging of AD (*r* = −0.37; *p* < 0.001, Pearson's coefficient). Furthermore, analysis of the CSF indicates that Jagged1 is present in different truncations and the bands above 130 KDa corresponding to the full length protein [HCTLs: 1 ± 0.25, AD: 0.24 ± 0.05; *t*_(10)_ = 2.93, *p* = 0.01; *n* = 6 patients per condition] and that below 130 KDa corresponding to the soluble protein [HCTLs: 1 ± 0.27, AD: 0.21 ± 0.03; *t*_(10)_ = 2.94, *p* = 0.02; *n* = 6 patients per condition] appear to be substantially reduced in 5 out of 6 AD patients examined as compared with healthy CTLs (Figures 1E,F). Analysis of the expression of DNER, a brain-specific Notch ligand (Eiraku et al., 2002; Kurisu et al., 2010), in CSF reveals no difference between conditions [HCTLs: 1 ± 0.37, AD: 0.78 ± 0.30; *t*_(10)_ = 0.98, *p* = 0.39; *n* = 6 patients per condition; Figures 1G,H]. Further analysis of CSF obtained by spinal tap from patients with MCI (MMSE: 21 ± 1.77) and healthy age-matched controls (MMSE:25.4 ± 2.23) reveals a tendency of Jagged1 to decrease in MCI [bands above 130 KDa; HCTLs: 1 ± 0.55, MCI: 0.22 ± 0.06; *t*_(8)_ = 1.66, *p* = 0.13; bands below 130 KDa; HCTLs: 1 ± 0.33, MCI: 0.40 ± 0.11; *t*_(8)_ = 1.76, *p* = 0.11; *n* = 5 patients per condition; Figure 1I and Figure S1B]. On the other hand, DNER's levels are constant in both MCI and Healthy CTLs [HCTLs: 1 ± 0.16, MCI: 0.85 ± 0.19; *t*_(8)_ = 0.64, *p* = 0.54; *n* = 5 patients per condition; Figure 1J and Figure S1C]. The progressive alteration of Jagged1 in the brain specimens and the CSF of AD patients suggests a specific and progressive imbalance in Jag1 availability in the CNS, which may be clinically relevant.

### Loss of Jagged1 affects spatial memory

**Figure 2:**
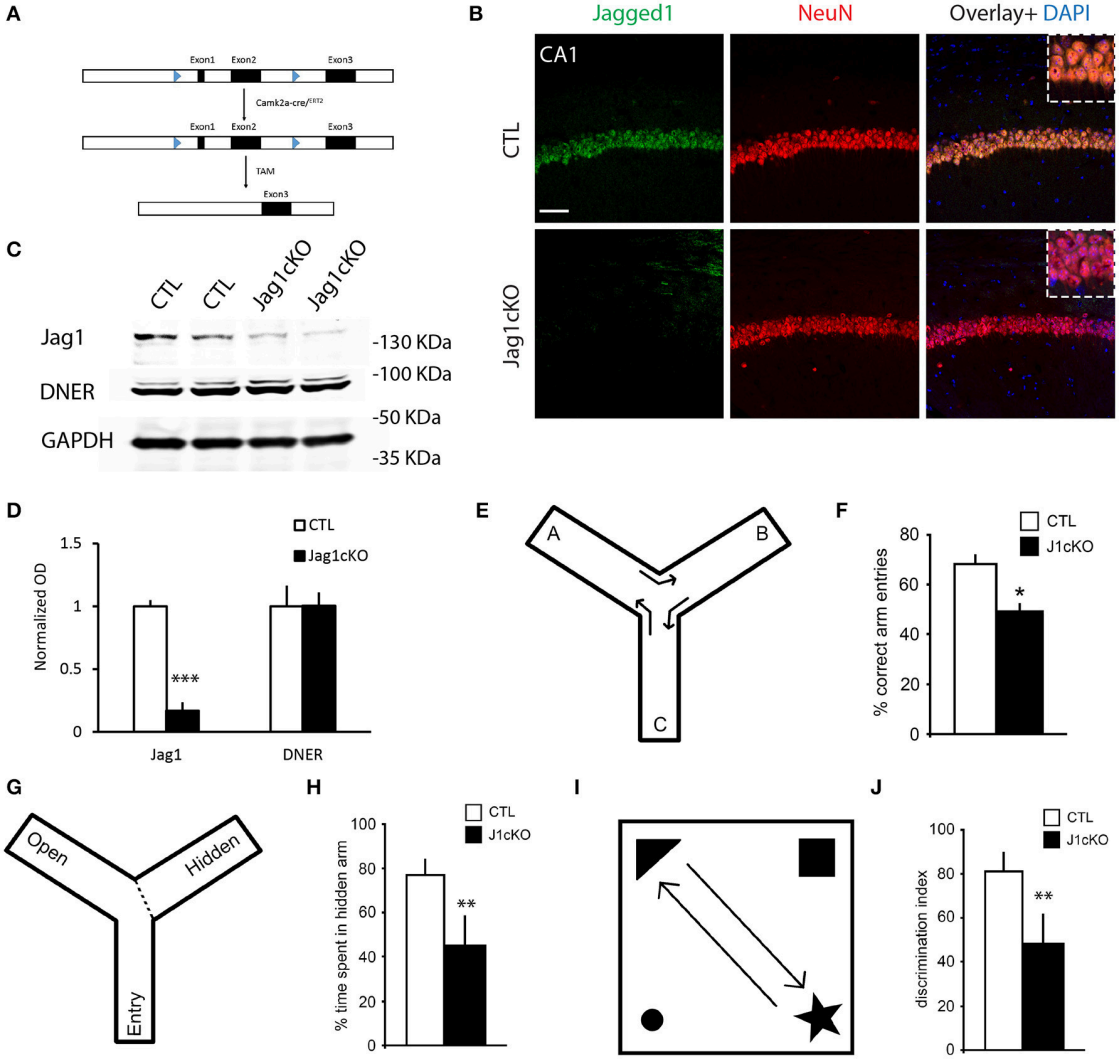
Targeted loss of Jagged1 in adult mouse neurons causes a spatial memory deficit. A schematic representation of the Jagged1 floxed allele used to generate the mice with TAM-inducible loss of Jagged1 gene **(A)**. Representative immunofluorescence images of single confocal z-plane showing a near complete loss of Jagged1 protein (green) from hippocampal pyramidal neurons labeled by NeuN (red) of Jagged1cKO mice **(B)**. Representative images from immunoblots showing the expression of Jagged1, DNER in Jagged1cKO mice and control mice **(C)**. GAPDH is used as a loading control. Bar graph showing the quantitation of optical densities of Jagged1 and DNER bands in CTLs and Jagged1cKO mice **(D)**. A graphic showing the behavioral arena for Y-maze spontaneous alternation test **(E)**. Jagged1cKO mice show a significant deficit in hippocampus dependent working memory in the Y-maze spontaneous alternation test **(F)**. A diagram showing the experimental arena for the hidden arm version of the Y-maze **(G)**. Jagged1cKO mice show a significant reduction in the time spent in the hidden arm, suggesting a spatial memory defect **(H)**. A schematic showing the experimental setup for the Novel Object Displacement test **(I)**. Jagged1cKO mice exhibit a statistical significant reduction in discrimination index, a measure of spatial memory defect **(J)**. Scale bar in **(B)** is 50 μm. Graphs are represented as mean ± SEM, ^*^p < 0.05, ^**^p < 0.01, and ^***^p < 0.001.

The reduction of Jagged1 in the brains of AD patients with severe dementia primed us to understand whether the loss of Jagged1 in neurons during adulthood is sufficient to induce memory loss and could be functionally correlated to memory decline in AD. We generated a Jagged1 conditional knockout mouse line (Jag1cKO) by crossing Jagged1^flox/flox^ (Nyfeler et al., 2005) with B6;129S6-Tg1Aibs/J (Camk2a-cre/^ERT2^) mice (Madisen et al., 2010). This strategy allowed us to obtain a neuron-specific deletion of Exon 1 and Exon 2 of Jagged1 gene in adult mice only after Tamoxifen (TAM) treatment (Figure 2A). This prevented any interference with prenatal or early-postnatal neurodevelopment. TAM application was performed in mice at 2–3 months of age and the loss of Jagged1 protein expression from hippocampal neurons was confirmed 10 days after TAM treatment. Using fluorescent immunohistochemistry for Jagged1 and NeuN, we observed a dramatic reduction in Jagged1 in hippocampal neurons as compared to Camk2α-cre/^ERT2^ injected with TAM (indicated as CTL; Figure 2B). Chromagen immunohistochemistry conferms the deletion in the CA fields of Jagged1 in TAM-injected Jag1cKO as compared to TAM-injected Camk2α-cre/^ERT2^ and uninjected Jag1cKO (Figure S2A). Western Blot analysis on whole brain tissue confirms a significant reduction (80%) of Jagged1 in the Jagged1cKO mice [CTLs: 1 ± 0.05, Jagged1cKO: 0.17 ± 0.07; *t*_(10)_ = 12.69, *p* < 0.001, *n* = 6 animals per group], whereas DNER appears unchanged [CTLs: 1 ± 0.21, Jagged1cKO: 1.01 ± 0.11; *t*_(10)_ = 0.21, *p* = 0.83, *n* = 6 animals per group; Figures 2C,D]. The robust loss in Jagged1 observable in the whole hippocampal tissue, is a strong indication that Jagged1 expression is mostly neuronal, as previously shown (Stump et al., 2002).

In order to correlate the loss of Jagged1 to memory decline, we tested memory performance in the Jagged1cKO and compared their performance to CTL mice. To exclude any interference from a possible anxiety phenotype or motor deficits on the memory task, we performed open-field and elevated plus maze tests. In the open-field, locomotion quantified by walking speed [CTLs: 34.55 ± 0.98 cm/s, Jagged1cKO: 37.23 ± 1.6 cm/s; *F*_(1, 8)_ = 2.29, *p* = 0.19, *n* = 4–5 animals per group] and anxiety behavior measured by the time spent in the center [CTLs: 317.37 ± 14.17 s, Jagged1cKO: 350.89 ± 21.57 s; *F*_(1, 8)_ = 0.99, *p* = 0.24, *n* = 4–5 animals per group]. To confirm the absence of an anxiety phenotype in the Jagged1cKO, we used the elevated plus-maze test and measured the time spent in the open arms [CTLs: 27.71 ± 5.09%, Jagged1cKO: 23.89 ± 9.45%; *F*_(1, 8)_ = 0.59, *p* = 0.47, *n* = 4–5 animals per group]. Both Jagged1cKOs and CTLs behave comparably as tested in the open field and elevated plus maze. We then investigated hippocampus-dependent memory by performing one-trial behavioral test for working memory (Y-maze alternation) (Figure 2E), and spatial reference memory (Y-maze hidden arm (Figure 2G) and Novel Object Displacement (Figure 2I). We used the Y-maze spontaneous alternation test that has been shown to be hippocampus-dependent (Sarnyai et al., 2000) (Figure 2E). The alternation in Jagged1cKO group (49.78 ± 2.55%) was close to chance (50%) in contrast to CTL mice [67.98 ± 3.75%; *F*_(1, 8)_ = 13.68, *p* = 0.01, *n* = 4–5 animals per group] (Figures 2E,F). The Y-maze hidden arm test (Alberi et al., 2011) showed a significant reference memory deficit in the Jagged1cKO mice as compared to CTL mice [ CTLs: 78.93 ± 4.16%, Jagged1cKO: 43.26 ± 14.2%; *F*_(1, 9)_ = 8.29, *p* = 0.02, *n* = 5 animals per group; Figures 2G,H]. We next used the NOD test (Mumby et al., 2002) to further validate the memory performance in the Jagged1cKO mice. We found that the lack of Jagged1 worsens the memory performance in the NOD test [CTLs: 81.67 ± 8.37%, Jagged1cKO: 48.5 ± 13.23%;*F*_(1, 11)_ = 12.72, *p* < 0.001, *n* = 6 animals per group; Figures 2I,J]. The behavioral tests demonstrate that Jagged1 expression in neurons is critical for spatial memory and suggest that reduction in Jagged1 expression may contribute to the memory deficit observed in AD patients.

### Jagged1 regulates activity-dependent notch signaling in direction of neural transmission

**Figure 3:**
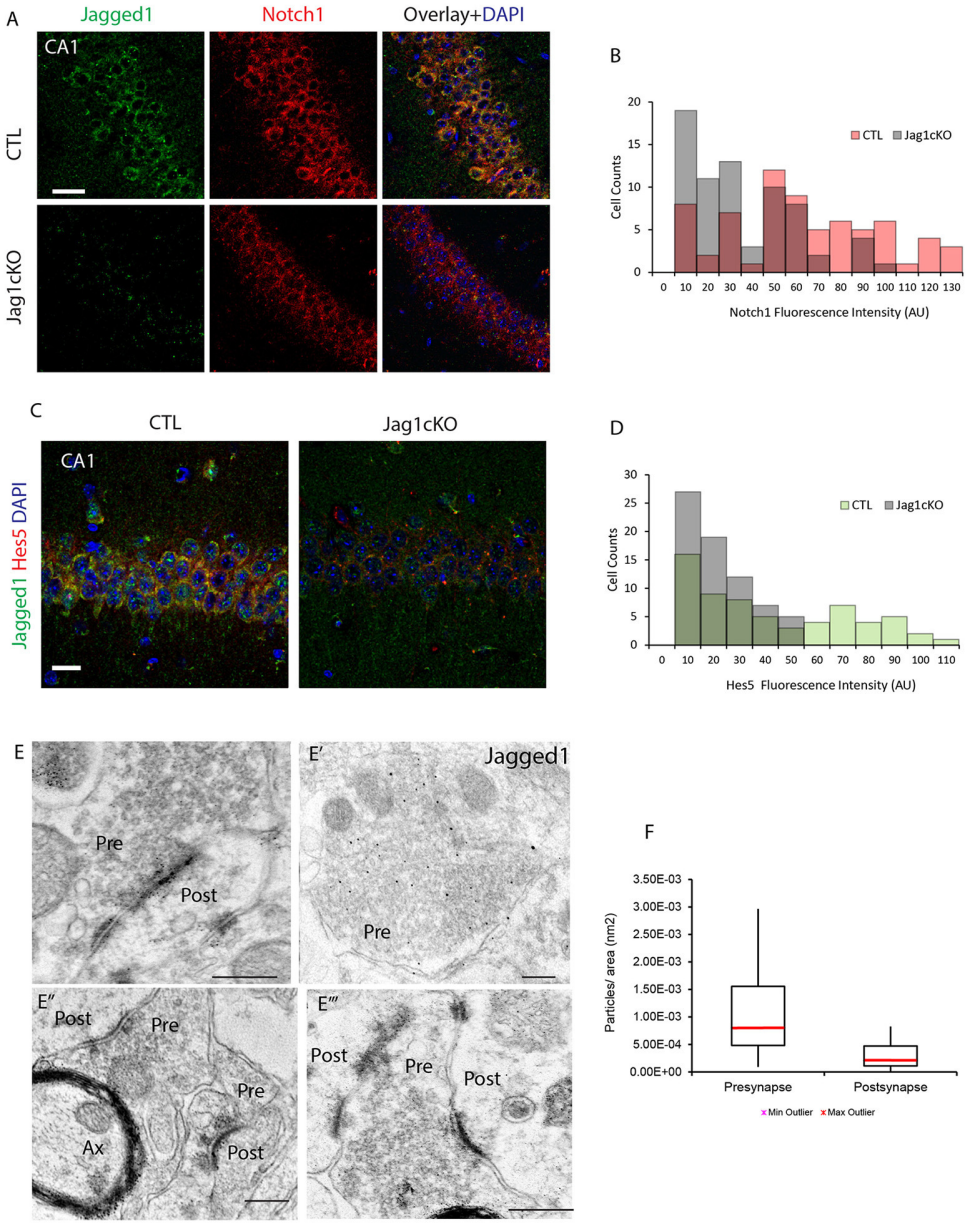
Jagged1 regulates learning-dependent Notch induction and is enriched presynaptically. Fluorescent double-labeling shows the expression of Notch1 (red) following spatial exploration in CTLs and Jagged1cKO **(A)**. Jagged1 labeling (green) is used to validate the absence of Jagged1 in the Jagged1cKO (Jag1cKOs) **(A,C)**. Diagram summarizing the Notch1 fluorescence intensities distribution in randomly picked 69 and 71 CA1 neurons in CTLs and Jagged1cKOs, respectively (*p* < 0.001) **(B)**. Double immunofluorescence of the Notch1 transcriptional target, Hes5, in CTLs and JaggedcKOs following spatial exploration **(C)**. Diagram summarizing the Hes5 fluorescence intensity distribution in randomly picked 64 and 70 CA1 neurons in CTL and Jagged1cKO, respectively (*p* < 0.001) **(D)**. Representatives gold Immuno-electron microscopy panels from three WT mice show that Jagged1 particles are localized in presynaptic terminals, bound to presynaptic vesicles (Pre) **(E–E‴)**. Immunogold particles are also apparent at the Postsynaptic density (Post) **(E,E″,E‴)**. Box Plot summarizing the particle counts in presynaptic and postsynaptic terminal areas drawn on Immunoelectromicroscopy micrograph for Jagged1 indicates that the presynapse is enriched with Jag1 particles as compared to the postsynaptic terminal (*p* < 0.001) **(F)**. Scale bars are: 20 μm in **(A)**, 10 μm in **(C)**, 400 nm in **(E–E‴)**. AU, arbitrary units; Pre, presynaptic; Post, postsynaptic; and Ax, axon.

To understand whether Jagged1 loss interferes with basal expression of Notch1, we performed chromagen immunolabeling on cage control TAM-injected Jag1cKO, TAM-injected CamKIIcreERT2, and uninjected Jag1cKO and did not observe any substantial difference (Figure S2B). Based on our previous work showing that spatial exploration induces Notch1 in CA field neurons (Alberi et al., 2011), we investigated whether the loss of Jagged1 would interfere with the typical exploration-induced increase in Notch1 in the CA fields. We performed double immunolabeling for Notch1 and Jagged1 and quantified the fluorescence intensity of Notch in a random selection of CA1 neurons. We observed that Notch1 expression was significantly lower in hippocampal ensembles of the CA1 field in Jagged1cKO mice as compared to CTL mice [CTLs, median: 53.57, IQR: 48.38, *U*_*CTL*_: 1,288; Jagged1cKO, median: 25.58, IQR: 35.13, *U*_*KO*_: 3,611, *p* < 0.001, *n* = 6 animals per group; Figure 3A]. The distribution of Notch1 intensities shows that in CTL mice the majority (74%) of counted neurons express high levels of Notch1 (50–120 AU) as opposed to Jagged1cKO (36%; Figure 3B). In order to ascertain whether Notch signaling activation is affected by the loss of Jagged1, we examined the expression of the transcriptional Notch target, Hes5 (Kopan and Ilagan, 2009), in CA1 fields following spatial exploration. Similar to Notch1, in the Jagged1cKO, Hes5 expression was significantly weaker as compared to CTL mice (CTLs, median: 28.52, IQR: 54.21, *U*_*CTL*_: 1,423; Jagged1cKO, median: 15.13, IQR: 18.46, *U*_*KO*_: 3,057, *p* < 0.001, *n* = 6 animals per group; Figure 3C). The distribution of Hes5 intensities shows that in Jagged1cKO mice all counted neurons (100%) express low levels of Hes5 (0–50 AU) as opposed to a CTL mice spanning the whole range of intensities (0–110 AU; Figure 3D). The results indicate that the loss of Jagged1 affects the induction of Notch1 expression and activity in ensembles of neurons.

To further assess whether Jagged1 modulates synaptic activity in direction of transmission in neurons, we used immuno-gold electron microscopy to visualize and quantify Jagged1 positive particles in the CA3-CA1 synapses. The Jagged1 immunoparticles appear localized to presynaptic vesicles and also concentrated at the synaptic junction (Figures 3E–E‴). The counting of gold particles on hippocampal synapses reveals that the presynaptic terminals are enriched in Jagged1 as compared to the postsynaptic side [presynaptic: 1.01 ^*^ 10^−3^ ± 4.36 ^*^ 10^−4^/ nm^2^, postsynaptic: 2.86 ^*^ 10^−4^ ± 1.36 ^*^ 10^−4^/ nm^2^; *F*_(1, 30)_ = 10.11, *p* = 0.003, *n* = 21 presynaptic and *n* = 21 postsynaptic areas, *n* = 3 wildtype mice; Figure 3F]. The presynaptic localization of Jagged1 and the postsynaptic enrichment of Notch1 (Brai et al., 2015) in hippocampal neurons suggests that the ligand and receptor are positioned in the direction of synaptic transmission and emphasize that Jagged1 may function as a presynaptic modulator of activity-dependent Notch signaling upon spatial learning.

## Discussion

The cell signaling pathways that influence neuronal activity and plasticity have received an increased attention in recent years. Further casting light on such signaling mechanism would allow us to devise better strategies against cognitive loss in progressive neurological diseases, such as AD. We have previously shown that a ubiquitous signaling pathway, such as Notch1, which is involved in cellular communication from development onward, is also essential for the formation of spatial memory (Alberi et al., 2011). In addition, Notch ligand Jagged1 influences synaptic potentiation (Wang et al., 2004) as well as memory formation (Sargin et al., 2013). This involvement of Notch signaling pathway in the process of memory formation seems to be conserved across species and may act via increasing phosphorylation of plasticity associated protein, CREB (Zhang et al., 2013; Brai et al., 2015). These results suggest that Notch pathway is an integral part of signaling networks involved in memory formation. Hence, it is tempting to speculate that the Notch signaling pathway may be compromised in patients with neurological disorders characterized by dementia, such as AD. Indeed, Notch signaling dysfunction has been implicated in sporadic as well as familial AD patients (Berezovska et al., 1998; Moehlmann et al., 2002; Brai et al., 2016). This decrease in Notch activity could stem from the misexpression of one or multiple Notch ligands. However, identity of such ligands had not been ascertained. The Notch ligand, Jagged1 has been shown to be expressed predominantly in neurons in the adult central nervous system (Stump et al., 2002; Alberi et al., 2011), and its constitutive downregulation results in severe memory deficits (Sargin et al., 2013). Interestingly, patients with Alagille syndrome, an autosomal dominant disorder caused by mutations in Jagged1 (Li et al., 1997; Ropke et al., 2003), show signs of mental retardation, besides systemic abnormalities. This further supports the involvement of Jagged1 in neuronal function through the Notch signaling cascade. Hence, in this paper, we investigated whether Jagged1 functions upstream of Notch signaling to regulate spatial memory, and its misexpression is involved in AD pathophysiology. Previous work from our laboratory indicated that Jagged1 and Notch1 are expressed in neurons in a stereotypical pattern with Jagged1 at presynaptic puncta and Notch1 at the postsynaptic terminal using primary neuronal cultures (Alberi et al., 2011). The immuno-electronmicroscopy for Jagged1 on fixed mouse hippocampal sections, presented in this paper, confirms that Jagged1 is predominantly located presynaptically and is associated to presynaptic vesicles. The presynaptic positioning of Jagged1 warrants ligand to receptor signaling in the direction of neural transmission, suggesting that an alteration in ligand availability may interfere with synaptic information exchange as in dementia (Huang et al., 2012). Indeed, we observe that in patients with severe dementia, Jagged1 expression is critically reduced (Figure 1), supporting the reduction in Notch activity in AD brains (Brai et al., 2016). Jagged1 labeling can also be found in fibrillary aggregates together with Notch1 likely as a result of neurites' break down. Moreover, the decrease of Jagged1 protein in the brain, as detected in the CSF of MCI and AD patients, appears to be gradual with the increasing severity of dementia (Figure 1). The specific implication of Jagged1 in Notch-dependent functions is emphasized by the evidence that the other Notch ligand, DNER, which is also expressed in hippocampal neurons (Kurisu et al., 2010), remains virtually unchanged with the progression of dementia (Figure 1). The alteration of Notch signaling components in AD underlines the potential involvement of this pathway in affecting synaptic transmission and emphasizes its role in memory decline.

Since the postnatal loss of Notch1 in the CA hippocampal fields (Alberi et al., 2011) and partial loss of Notch1, RBPJK and Jagged1 in heterozygous mice (Sargin et al., 2013) results in a learning and memory deficit, we next addressed whether Jagged1 misexpression is sufficient to induce memory decline. In order to knockout Jagged1 gene during adulthood, we have devised a conditional KO mouse for Jagged1, using TAM inducible Cre (Jagged1cKO) (Figure 2). The advantage of this model as compared to the one used by Sargin (Sargin et al., 2013) is that gene deletion is induced only in the adult brain and it is nearly complete (80%), eliminating any confounding developmental effects of residual Jagged1 function. This also indicates that the majority of Jagged1 is expressed in neurons as opposed to other cell types in the brain. Similarly to the Notch1cKOs, Jagged1cKO display a spatial reference memory deficit in the Y-maze and NOD tests (Figure 2). Using a different battery of tests for spatial memory, Sargin and colleagues also found a deficit in spatial memory formation in the Jagged1^+/−^ mice. This emphasizes that Jagged1, comparably to Notch1, is required for spatial memory in adult mice and that dosage of the ligand may be critical for activity-dependent Notch signaling. To further resolve the functional correlation between Jagged1 and Notch1 activity in spatial learning and memory, we showed that the loss of Jagged1 affects the learning-dependent induction of Notch1 expression and its activity, as indicated by the absence of CA1 neurons expressing detectable levels of Hes5 (Figure 3). The loss of Jagged1 not only interferes with Notch pathway activation but may affect the known positive feedback regulation of DSL ligands on the signal sending neuron, depleting further the synapse of putative Notch ligands (D'Souza et al., 2008). Thus, Jagged1 appears to regulate Notch-dependent plasticity in an activity-dependent manner. This is in line with previous data showing that Jagged1 expression and Notch1 signaling are induced by an increase in synaptic activity (Alberi et al., 2011) and is further supported by evidence that a soluble peptide of Jagged1 can potentiate LTP (Wang et al., 2004) and activate Notch1 in neuronal cultures (data not shown). The evidence that in the setting of neurons a soluble form of Jagged1 can induce Notch1 pathway activation is in stark contrast to the reported requirement in stem cells of a membrane bound ligand to exercise a pulling force on the extracellular portion of the Notch to activate the receptor (Hansson et al., 2010). However, a previous report showed that the shedding of Jagged1 from the cell signaling cell is induced by the interaction with Notch and is mediated by ADAM-17 (LaVoie and Selkoe, 2003). Likewise in neurons, Jagged1 may exist both in a membrane-bound and soluble form. Indeed, from our IEM data, it appears that Jagged1 is present in presynaptic vesicles (Figure 3), and may be deployed upon synaptic stimulation, similar to a neurotransmitter. In condition of elevated synaptic activity, it is conceivable that the soluble ligand activates Notch in the postsynaptic compartment, where the receptor is enriched (Brai et al., 2015), and Notch1 processing results from the clustering of the endosomal trafficking molecule Arc/Arg3.1 to the γ-secretase complex (Alberi et al., 2011; Wu et al., 2011). To this effect, we show that either the loss of Jagged1 or Arc/Arg3.1 can affect learning-dependent Notch activation and result in a spatial memory deficit. In light of these results, the observed reduction of Jagged1 in AD may cause a reduction in Notch1 activity, underlying the memory decline.

Altogether, this work expands our understanding of the role of Notch signaling in learning and memory and emphasizes the relevance of presynaptic Jagged1 ligand in neurons in potentiating neuronal Notch activity. This work suggests that modulation of the pathway, through Jagged1 application, could be a therapeutically viable approach to counteract cognitive decline in dementia.

## Author contributions

SM: Performed the experiments in rodents and designed the Jag1cKO mouse line, performed the data analysis and wrote part of the manuscript. MJ: Performed the experiments on the human specimen. JA: Provided the *ex-vivo* CSF samples and performed the clinical diagnosis of the MCI and healthy control patients. LA designed the study and supervised the writing of the manuscript.

### Conflict of interest statement

The authors declare that the research was conducted in the absence of any commercial or financial relationships that could be construed as a potential conflict of interest.

## Supplementary material

Figure S1 Severe brain atrophy of AD specimen and pattern of Jagged1 and DNER expression in CSF of MCI and Healthy control patients. Representative DAPI staining of enthorinal sections from healthy CTL and AD patient show the substantial atrophy of the hippocampal (HP) and cortical (EC) regions as well as the enlargement of the ventricle (V) in a AD patient as compared to a Healthy control **(A)**. Immunoblotting on CSF from 5 MCI and 5 Healthy control patients reveals that Jagged1 bands >130 KDa (blue stars) corresponding to the full length and the and <130 KDa (red stars) indicating the soluble Jagged1 tend to be less represented in the CSF from AD patients **(B)**. Representative DNER immunoblotting of the CSF from the same patients shows no differennce in the expected band <100 KDa (blue stars) **(C)**. Scale bar is 5 mm.

Figure S2 Jagged1 and Notch1 basal expression in TAM-injected and -uninjected transgenic mice. Representative Jagged1 chromagen immunohistochemistry on saggital sections shows the loss of Jagged1 expression in the hippocampal CA fields of the TAM-injected Jag1cKO as compared to TAM-injected CamKIIcre ERT2 mice and uninjected Jag1cKO **(A)**. Chromagen immunolabeling showing the expression of Notch1 in the three mouse lines used in the study **(B)**. Scale bars in **(A,B)** is 5 mm.
