# Supplementary figures and images for: Jagged1 Is Altered in Alzheimer's Disease and Regulates Spatial Memory Processing
Source: Front Cell Neurosci. 2017 Aug 9;11:220. doi: 10.3389/fncel.2017.00220 (PMC5552758; doi:10.3389/fncel.2017.00220)

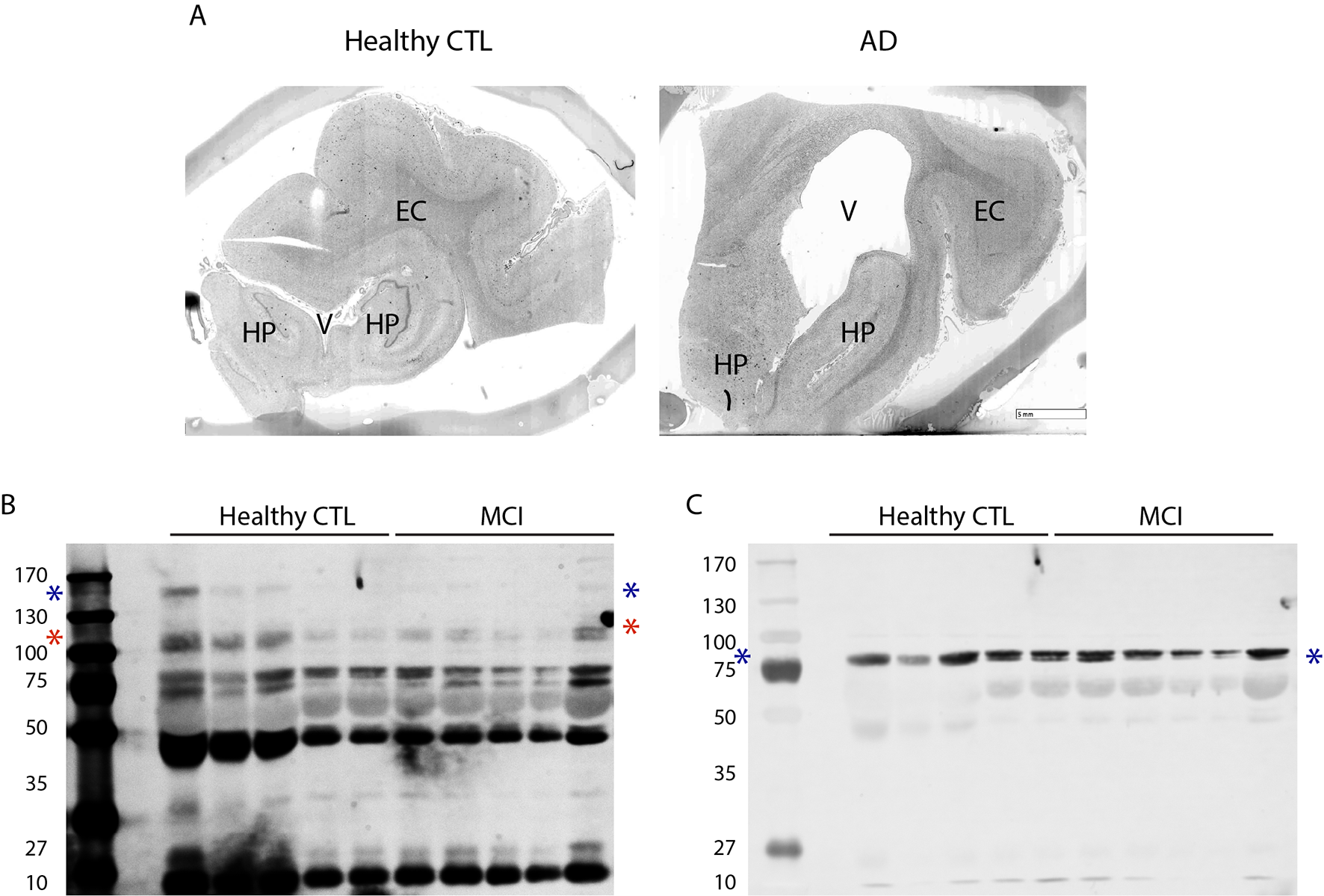

Supplement: Figure S1 — Severe brain atrophy of AD specimen and pattern of Jagged1 and DNER expression in CSF of MCI and Healthy control patients. Representative DAPI staining of enthorinal sections from healthy CTL and AD patient show the substantial atrophy of the hippocampal (HP) and cortical (EC) regions as well as the enlargement of the ventricle (V) in a AD patient as compared to a Healthy control (A). Immunoblotting on CSF from 5 MCI and 5 Healthy control patients reveals that Jagged1 bands >130 KDa (blue stars) corresponding to the full length and the and <130 KDa (red stars) indicating the soluble Jagged1 tend to be less represented in the CSF from AD patients (B). Representative DNER immunoblotting of the CSF from the same patients shows no differennce in the expected band <100 KDa (blue stars) (C). Scale bar is 5 mm. [file Image1.TIF]

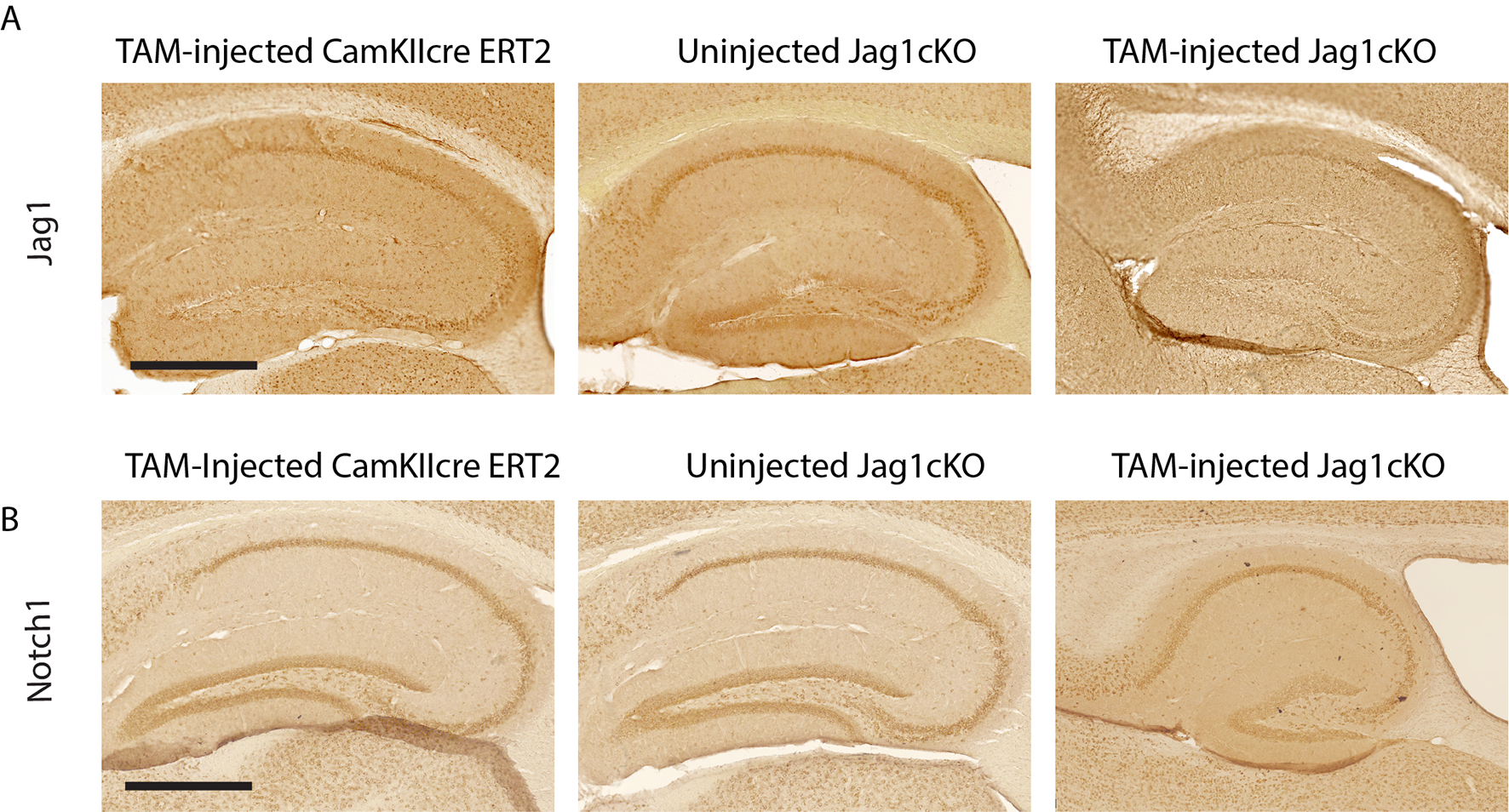

Supplement: Figure S2 — Jagged1 and Notch1 basal expression in TAM-injected and -uninjected transgenic mice. Representative Jagged1 chromagen immunohistochemistry on saggital sections shows the loss of Jagged1 expression in the hippocampal CA fields of the TAM-injected Jag1cKO as compared to TAM-injected CamKIIcre ERT2 mice and uninjected Jag1cKO (A). Chromagen immunolabeling showing the expression of Notch1 in the three mouse lines used in the study (B). Scale bars in (A,B) is 5 mm. [file Image2.tif]
